# Dual mutations in the whitefly nicotinic acetylcholine receptor *β1* subunit confer target-site resistance to multiple neonicotinoid insecticides

**DOI:** 10.1371/journal.pgen.1011163

**Published:** 2024-02-20

**Authors:** Cheng Yin, Andrias O. O’Reilly, Shao-Nan Liu, Tian-Hua Du, Pei-Pan Gong, Cheng-Jia Zhang, Xue-Gao Wei, Jing Yang, Ming-Jiao Huang, Bu-Li Fu, Jin-Jin Liang, Hu Xue, Jin-Yu Hu, Yao Ji, Chao He, He Du, Chao Wang, Rong Zhang, Qi-Mei Tan, Han-Tang Lu, Wen Xie, Dong Chu, Xu-Guo Zhou, Ralf Nauen, Lian-You Gui, Chris Bass, Xin Yang, You-Jun Zhang

**Affiliations:** 1 State Key Laboratory of Vegetable Biobreeding, Institute of Vegetables and Flowers, Chinese Academy of Agricultural Sciences, Beijing, P. R. China; 2 Hubei Engineering Technology Center for Pest Forewarning and Management, College of Agriculture, Yangtze University, Jingzhou, Hubei, P. R. China; 3 School of Biological & Environmental Sciences, Liverpool John Moores University, Liverpool, United Kingdom; 4 Hunan Provincial Key laboratory of Pesticide Biology and Precise Use Techology, Hunan Agricultural Biotechnology Research Institute, Changsha, P. R. China; 5 Key Laboratory of Integrated Crop Pest Management of Shandong Province, School of Agriculture and Plant Protection, Qingdao Agricultural University, Qingdao, Shandong Province, China; 6 Department of Entomology, University of Kentucky, Lexington, Kentucky, United States of America; 7 Bayer AG, Crop Science Division, R&D, Monheim, Germany; 8 Centre for Ecology and Conservation, University of Exeter, Penryn Campus, Penryn, Cornwall, United Kingdom; The University of North Carolina at Chapel Hill, UNITED STATES

## Abstract

Neonicotinoid insecticides, which target insect nicotinic acetylcholine receptors (nAChRs), have been widely and intensively used to control the whitefly, *Bemisia tabaci*, a highly damaging, globally distributed, crop pest. This has inevitably led to the emergence of populations with resistance to neonicotinoids. However, to date, there have been no reports of target-site resistance involving mutation of *B*. *tabaci* nAChR genes. Here we characterize the nAChR subunit gene family of *B*. *tabaci* and identify dual mutations (A58T&R79E) in one of these genes (*BTβ1*) that confer resistance to multiple neonicotinoids. Transgenic *D*. *melanogaster*, where the native nAChR *Dβ1* was replaced with *BTβ1*^A58T&R79E^, were significantly more resistant to neonicotinoids than flies where *Dβ1* were replaced with the wildtype *BTβ1* sequence, demonstrating the causal role of the mutations in resistance. The two mutations identified in this study replace two amino acids that are highly conserved in >200 insect species. Three-dimensional modelling suggests a molecular mechanism for this resistance, whereby A58T forms a hydrogen bond with the R79E side chain, which positions its negatively-charged carboxylate group to electrostatically repulse a neonicotinoid at the orthosteric site. Together these findings describe the first case of target-site resistance to neonicotinoids in *B*. *tabaci* and provide insight into the molecular determinants of neonicotinoid binding and selectivity.

## Author summary

The sweet potato whitefly, *Bemisia tabaci*, is an important target pest of neonicotinoid insecticides. Much is known about the neonicotinoid-resistance evolved in whitefly by over-expressed detoxifying enzymes. But the toxicological targets of neonicotinoids, the nAChR subunit genes in whitefly are less well understood. In this study, we characterized the nAChR subunit gene family of whitefly, and identified dual mutations in beta1 subunit that confer resistance to multiple neonicotinoid insecticides. These findings are significant as they demonstrate how resistance can arise as a result of mutations in insects that had a combined effect to repulse the insecticides thus overcoming the insect selectivity of an important class of insecticides.

## Introduction

Neonicotinoids are effective and mammalian-safe insecticides, that play an important role in crop protection and animal health [1]. They target insect nicotinic acetylcholine receptors (nAChRs) that transmit fast synaptic signals in insect nervous systems [2,3]. nAChRs are comprised of five subunits, with key ligand-binding regions built from loops in the N-terminal extracellular region of two neighboring subunits [4]. Positively charged residues present only in insect nAChR subunits of these loop regions have been shown to be important determinants of neonicotinoid selectivity for insects, for their electrostatic interaction with the negative nitro or cyano groups of neonicotinoids [5]. Subunits are characterized as α or non-α subunits by the presence or not of the two adjacent cysteine residues in loop C that required for ACh binding [6]. The nAChR subunit gene family of insects typically comprises around 10 genes [6–12]. In the case of the model insect, *Drosophila melanogaster*, *Dα1*, *Dα2*, *Dβ1*, and *Dβ2* have been shown to play an important role in the insecticidal activity of neonicotinoids [13,14]. However, the relative importance of different nAChR subunit genes in non-model insect species is much less well understood.

The evolution of insecticide resistance is a serious problem that can severely compromise the efficacy of insecticides and threatens global food security and our ability to control numerous insect disease vectors [15]. Research on the genetic basis of resistance to neonicotinoids in pest insects has shown that resistance to this insecticide class most commonly involves the enhanced expression or activity of detoxification enzymes such as cytochromes P450 [15–21]. In contrast, mutations in nAChR genes that lead to target-site resistance to neonicotinoids have been much less frequently described. Indeed, to date, only two examples of functionally characterized target-site mutations that confer resistance to neonicotinoids have been described in insect pests. The first of these is the Y151S mutation in *Nlα1* or *Nlα3* of the brown planthopper, *Nilaparvata lugens*, that was shown to confer resistance to imidacloprid in a laboratory insecticide-selected strain [22]. Subsequently, the R81T mutation has been identified in the *β1* subunit of the aphids *Myzus persicae* and *Aphis gossypii* in field-collected imidacloprid-resistant strains [23–25].

The whitefly, *Bemisia tabaci*, is a highly destructive pest with a worldwide distribution [26]. Due to its broad host range and its ability to spread numerous plant viruses, it often causes great losses to agriculture production. Control of this pest primarily depends on chemical insecticides at present, especially the neonicotinoids [16]. Research on the mechanisms of resistance to neonicotinoids in this species has frequently implicated the over-expression of detoxification enzymes, especially cytochromes P450 [27–30]. To date, the nAChR subunit gene family of *B*. *tabaci* has not been characterized, and no case of target-site resistance to neonicotinoids caused by genetic variants in nAChR genes has been described.

In this study, we characterize the nAChR subunit gene family of *B*. *tabaci* and identify mutations in one of these genes that are associated with resistance to neonicotinoids. We use a range of post-genomic functional and computational approaches to understand how these mutations confer resistance and gain insight into neonicotinoid selectivity and mode of action.

## Results

### The nAChR gene family of *Bemisia tabaci*

**Fig 1:**
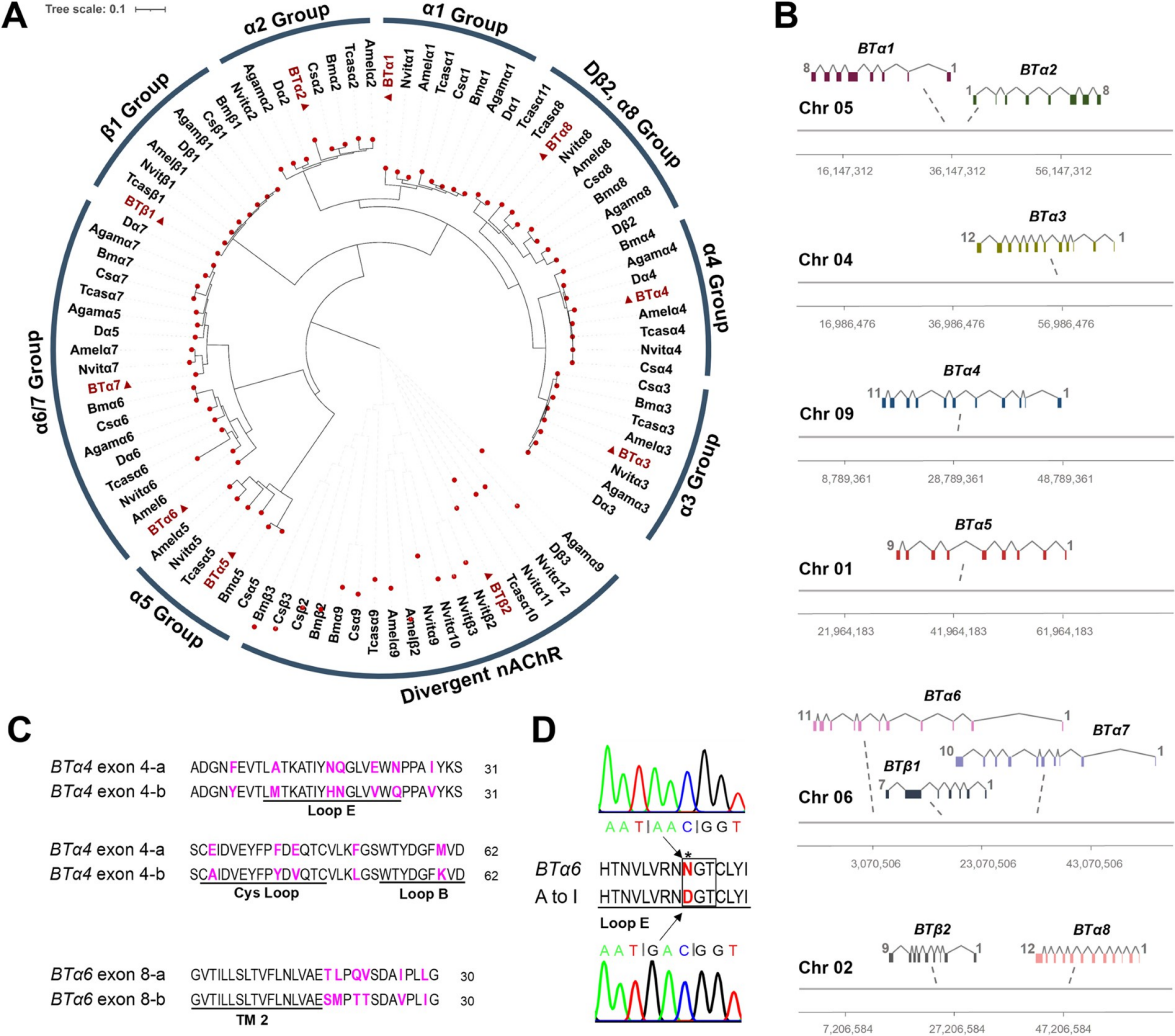
The nAChR family of *B*. *tabaci*. (**A**) Molecular phylogenetic analysis of nAChRs in *B*. *tabaci* and other insects. Species codes are Agam = *Anopheles gambiae*, Amel = *Apis mellifera*, Bm = *Bombyx mori*, BT = *Bemisia tabaci*, Cs = *Chilo suppressalis*, D = *Drosophila melanogaster*, Nvit = *Nasonia vitripennis*, Tcas = *Tribolium castaneum*. The amino acid sequences of insect nAChRs were aligned using Clustal W. A phylogenetic tree was generated in MEGA 7 using the neighbor-joining method with 1000 bootstrap replicates. (**B**) Gene structures and chromosomal locations of *B*. *tabaci* nAChRs. (**C**) Sequences of alternative exons in *BTα4* and *BTα6*. Amino acids that changed are highlighted. (**D**) A to I editing site in *BTα6*. The N-glycosylation site is boxed.

From the *B*. *tabaci* genome database [31], we identified and cloned ten candidate nAChR genes. Eight of these subunits can be characterized as α type subunits and two as non-α type subunits based on the presence or absence of adjacent cysteine residues in loop C (S1 Fig) [6]. A phylogenetic comparison of these nAChR subunit genes with those of other insects, reveal that nine of the *B*. *tabaci* nAChR genes have orthologues in other insects [6–12], allowing us to assign them as *α1–8* subunit genes (*BTα1–8*) and a *β1* subunit gene (*BTβ1*) (Figs 1A and S2). The remaining non-α subunit of *B*. *tabaci* has no conserved orthologue in other insects but rather belongs to the divergent subunit group of insect nAChRs (*BTβ2*) (Figs 1A and S2) [32]. The nAChR subunit genes identified in this study are distributed across the genome of *B*. *tabaci*, occurring on chromosomes 1, 2, 4, 5, 6 and 9, with only *BTα1* and *BTα2* clustered within 150 kb of each other on chromosome 5 (Fig 1B and S1 Table). Cloning and sequencing were used to validate the gene sequences of the ten *B*. *tabaci* nAChR subunit genes and revealed that *BTα4* and *BTα6* have alternatively spliced exons (Fig 1C). For *BTα4*, the alternative splicing occurs at exon 4, and results in alternative amino acid sequences in regions encoding Loop B, Loop E and the Cys loop. For *BTα6*, alternative versions of exon 8, result in divergent amino acid sequence for the region between transmembrane domains 2 and 3 (TM 2 and TM 3). A to I editing that causes substitution of amino acid was only observed in *BTα6* from the analysis of sequences in S1 Dataset, and was further confirmed by sequencing the gDNA sequences from individuals of strains in S2 Dataset. In this case, one amino acid in an N-glycosylation consensus site of Loop E region was altered from N (AAC) to D (GAC) (Fig 1D and S2 Dataset).

### Neonicotinoid resistance is not mediated by metabolic mechanisms but is associated with mutations in the *BTβ1* genes in a *B*. *tabaci* field strain

**Fig 2:**
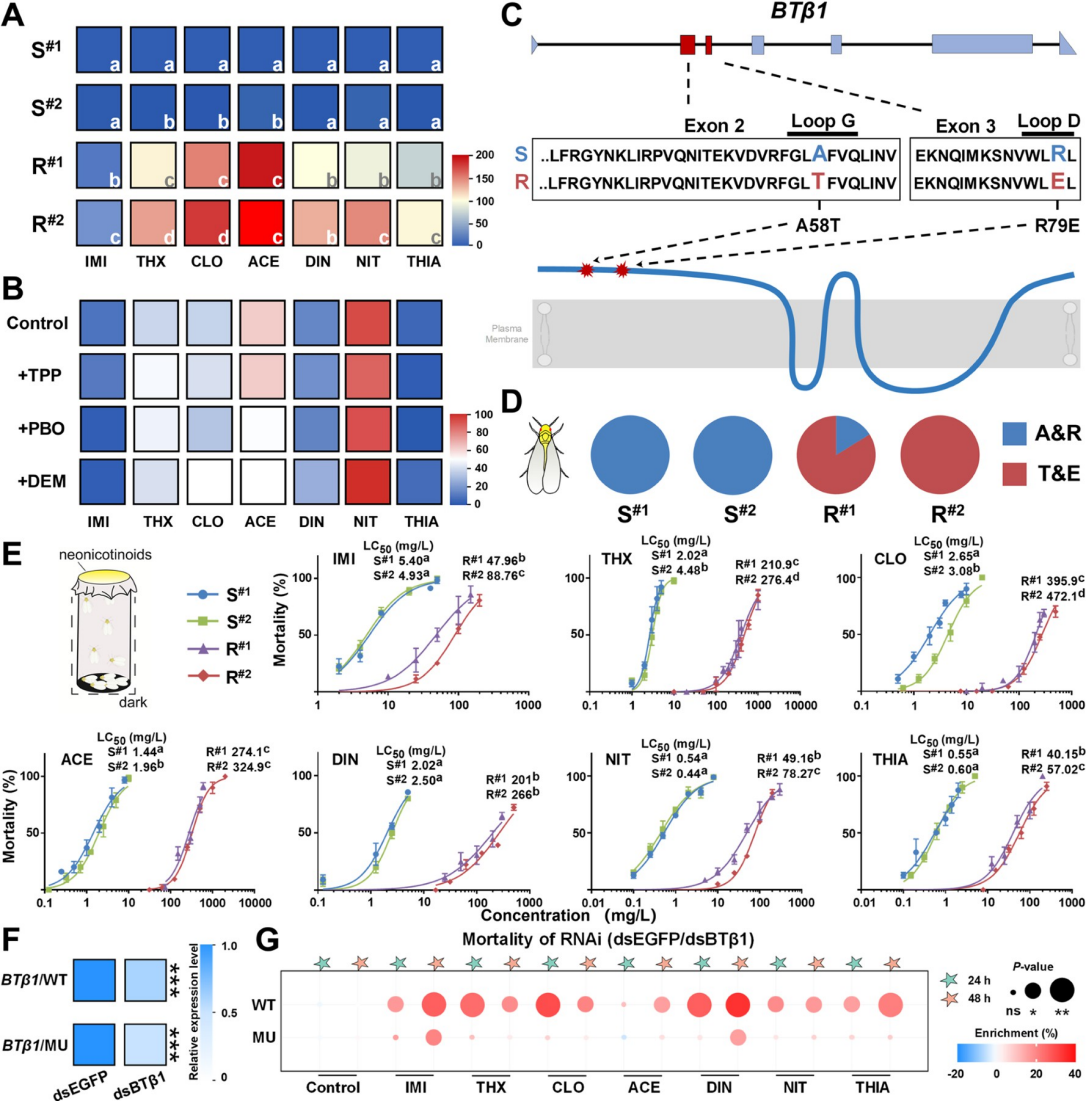
The A58T and R79E mutations in *BTβ1* of a neonicotinoid resistant *B*. *tabaci* MEAM1 strain. (**A**) Resistance levels of the *B*. *tabaci* strains to seven neonicotinoid insecticides. IMI, imidacloprid; THX, thiamethoxam; CLO, clothianidin; ACE, acetamiprid; DIN, dinotefuran; NIT, nitenpyram; THIA, thiacloprid. Values were generated by calculating the LC_50_ value of each strain to each insecticide and comparing to those of the S^#1^ strain. Different letters on heat map represent significant difference between their LC_50_ values for each insecticide (*P* < 0.05). (**B**) Mortalities of mutant *B*. *tabaci* strain in synergism bioassays. Values are means of three biological replicates (*n* = 3). Whiteflies fed insecticide without synergist were used as control. (**C**) Location of the A58T and R79E mutations in *BTβ1*. Loop G region in exon 2 and Loop D region in exon 3 are indicated by the black line above. (**D**) Frequency of A58T&R79E in neonicotinoid susceptible and resistant strains. S^#1^ and S^#2^ are two laboratory neonicotinoid susceptible strains, R^#1^ is a neonicotinoid resistant field strain, and R^#2^ was a homozygous mutant strain derived from R^#1^. Sixty individuals of each strain were genotyped. (**E**) Nonlinear response curve of the mortality of *B*. *tabaci* strains against log-doses of different neonicotinoids. Values are mean ± SEM (*n* = 3). Different letters on LC_50_ values represent significant difference (*P* < 0.05). (**F**) Relative expression levels of *BTβ1* after feeding adult whiteflies dsBTβ1. Adult whiteflies fed on dsEGFP were used as control. Values are means of three biological replicates (*n* = 3), asterisks represent significant difference between the treatments and controls (***P < 0.001). (**G**) Difference in mortality of *B*. *tabaci* strains fed dsEGFP or dsBTβ1 and exposed to different insecticides. Values are means of three biological replicates (*n* = 3). Circles of different size represent significant difference (ns, no significant difference; *, *P* < 0.05; **, *P* < 0.01).

A strain of *B*. *tabaci* MEAM1 (Middle East-Asia Minor 1, B biotype) (designated R^#1^) collected from Xinjiang, Urumqi (43° 82’ N, 87° 58’ E) was found to exhibit broad cross-resistance to different neonicotinoid compounds in insecticide bioassays. Specifically, comparison of the lethal concentration 50% (LC_50_) values obtained for this strain with those of an insecticide susceptible *B*. *tabaci* MEAM1 strain (S^#1^) resulted in resistance ratios of 8.88-fold for imidacloprid, 104.51-fold for thiamethoxam, 149.4-fold for clothianidin, 191.01-fold for acetamiprid, 99.41-fold for dinotefuran, 91.72-fold for nitenpyram and 72.46-fold for thiacloprid (Fig 2A and S2 Table).

To investigate the role of metabolic mechanisms in the resistance of the R^#1^ strain to neonicotinoids, insecticide bioassays were conducted in combination with inhibitors of detoxification enzymes frequently implicated in resistance, including the cytochrome P450 inhibitor piperonyl butoxide (PBO), the glutathione S-transferase inhibitor diethyl maleate (DEM), and the esterase inhibitor triphenyl phosphate (TPP). No significant increase in mortality of the resistant strain was observed when these inhibitors were used in combination with neonicotinoid insecticides compared to the insecticide alone control (Fig 2B), suggesting the resistance of this strain is not mediated by the most common metabolic mechanisms.

To investigate the role of target-site mutation in the resistance of the R^#1^ strain the ten nAChR subunit genes identified in this study were cloned and compared between this strain, S^#1^, and a second laboratory insecticide-susceptible strain (S^#2^) that exhibited a similar level of sensitivity to neonicotinoids as S^#1^, while LC_50_ values of thiamethoxam, clothianidin and acetamiprid showed statistical difference (*P* < 0.05) (Fig 2A and S2 Table). No nonsynonymous mutations were observed in the sequences obtained that consistently distinguish the resistant and susceptible strains in the *BTα1–8* and *BTβ2* genes (S1 Dataset). However, two linked mutations were identified in the *BTβ1* gene of the R^#1^ strain, that were not present in the S^#1^ and S^#2^ strains resulting in the amino acid substitutions A58T (GCT → ACT) and R79E (CGA → GAA), located in the second and third exons of *BTβ1*, respectively (Fig 2C and S1 Dataset). Screening of 60 individuals of each strain at the mutation sites confirmed these findings and revealed that the two mutations are present at a frequency of 83.82% in the R^#1^ strain (Fig 2D and S3 Dataset).

To facilitate further investigation of the role of these candidate mutations in resistance, a *B*. *tabaci* strain (R^#2^) was created that was homozygous for the two mutations by crossing pairs of newly emerged male and female individuals from R^#1^ (S3 Dataset). Insecticide bioassays of this strain revealed increased resistance to all neonicotinoid insecticides compared to the R^#1^ strain (Fig 2E), with the resistance level to neonicotinoids (relative to S^#1^) increased, from 8.88-fold to 16.44-fold for imidacloprid, 104.51-fold to 136.97-fold for thiamethoxam, 149.4-fold to 178.15-fold for clothianidin, 191.01-fold to 226.41-fold for acetamiprid, 99.41-fold to 131.55-fold for dinotefuran, 91.72-fold to 146.02-fold for nitenpyram, and 72.46-fold to 102.91-fold for thiacloprid (Fig 2A and S2 Table). However, LC_50_ values of acetamiprid and dinotefuran were not statistically different between R^#1^ and R^#2^ strain (Fig 2A and S2 Table). Relative expression levels of 13 genes that previously implicated in neonicotinoid resistance in whitefly, the *CYP6CM1*, *CYP6CX1*, *CYP6CX3*, *CYP6CX4*, *CYP6CX5*, *CYP6DZ4*, *CYP6DZ7*, *CYP4C1*, *CYP4G68*, *CYP4C64*, *GSTd7*, *GST14* and *ABCG3* [18–20,30] were detected to confirm they are not involved in the resistance formation in R^#2^ strain (S3 Fig).

We previously demonstrated that knock down of *BTβ1* expression using RNA interference can decrease the susceptibility of *B*. *tabaci* to neonicotinoids [33]. To examine if neonicotinoid susceptibility can also be influenced by knocking down the mutant *BTβ1*^A58T&R79E^ gene we fed double-stranded RNA transcribed from a conserved region of *BTβ1* to the S^#1^ and R^#2^ strains. The expression level of *BTβ1* and *BTβ1*^A58T&R79E^ was significantly reduced by 39.4% (p = 1.64 × 10^−5^) and 43.8% (p = 6.68 × 10^−4^) in the S^#1^ and R^#2^ strains respectively (Fig 2F), following feeding on dsBTβ1 compared to the dsEGFP control. Consistent with our previous findings, knockdown of *BTβ1* in the wildtype S^#1^ strain resulted in significant decreases in sensitivity to neonicotinoids compared to the dsEGFP group at 24h and 48h, with reductions in mortality of 15.17% at 24 h (p = 2.01 × 10^−2^) and 23.84% at 48 h (p = 4.69 × 10^−3^) for imidacloprid, 21.53% (p = 3.99 × 10^−3^) and 16.89% (p = 2.16 × 10^−2^) for thiamethoxam, 15.73% at 48 h for acetamiprid (p = 1.47 × 10^−2^), 25.4% (p = 1.49 × 10^−4^) and 24.57% for dinotefuran (p = 3.35 × 10^−4^), 15.18% (p = 1.49 × 10^−2^) and 16.26% (p = 1.73 × 10^−2^) for nitenpyram, and 14.17% (p = 2.57 × 10^−2^) and 21.48% (p = 1.52 × 10^−4^) for thiacloprid (Fig 2G). In contrast, knockdown of *BTβ1*^A58T&R79E^ in the mutant R^#2^ strain resulted in decreased sensitivity to just two neonicotinoid insecticides, 16.66% for imidacloprid (p = 3.09 × 10^−2^) and 16.14% for dinotefuran at 48 h (p = 1.39 × 10^−2^) (Fig 2G).

### Transgenic flies expressing *BTβ1*^A58T&R79E^ exhibit resistance to neonicotinoids

**Fig 3:**
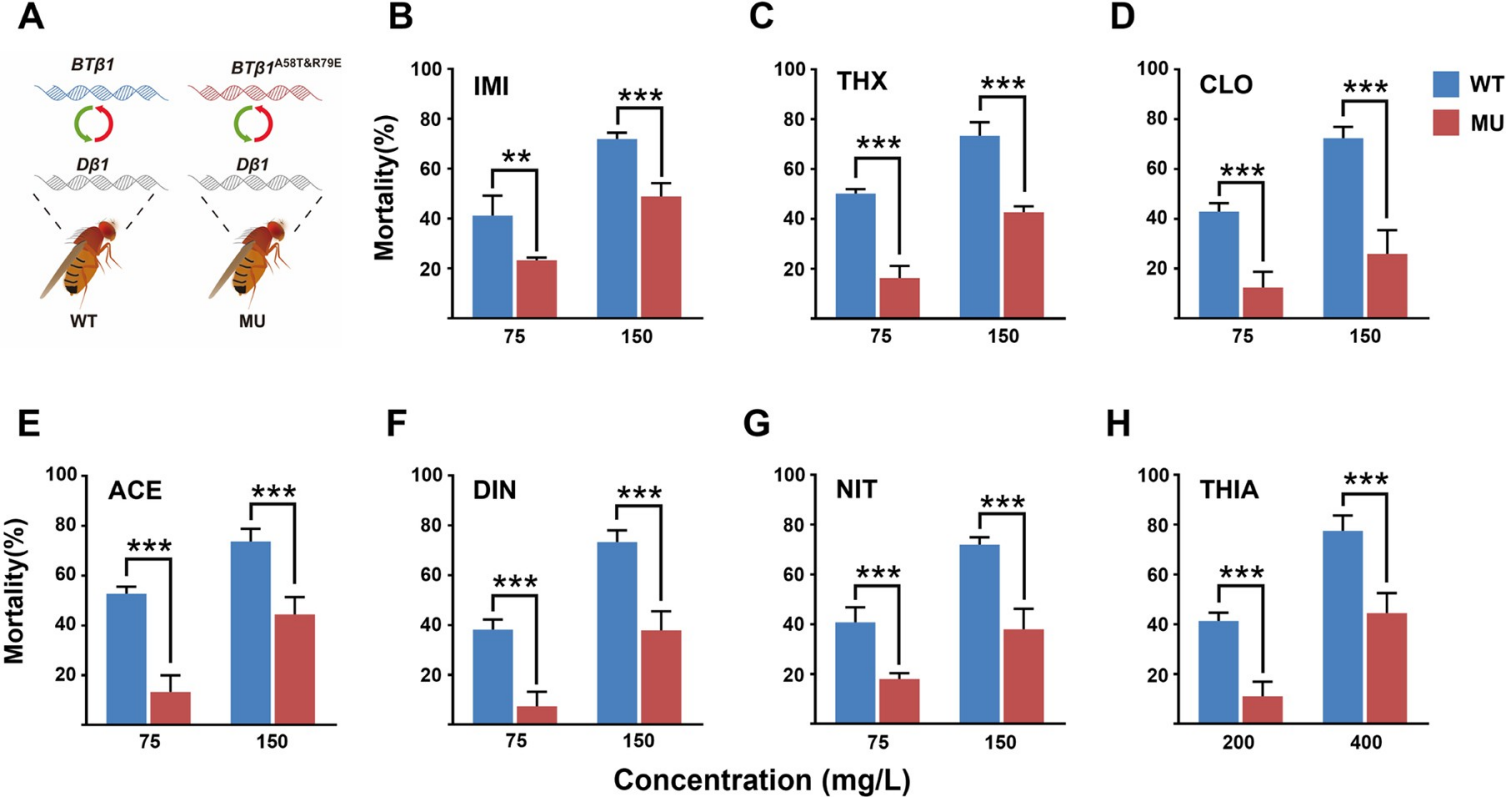
Sensitivity of transgenic *D*. *melanogaster* to seven neonicotinoid insecticides where native *Dβ1* was replaced with *BTβ1* or *BTβ1*^A58T&R79E^. (**A**) A schematic of the process used to generate transgenic flies. (**B-H**) Sensitivity of *BTβ1* or *BTβ1*^A58T&R79E^ expressing *D*. *melanogaster* to seven neonicotinoid insecticides. Values are mean ± SEM (*n* = 3), each bar represents the mean ± standard deviation, asterisks represent significant differences in between the treatments and controls (**P < 0.01, ***P < 0.001).

To validate the role of A58T and R79E in neonicotinoid resistance we constructed transgenic strains of *Drosophila melanogaster* by substituting the *Dβ1* gene with *BTβ1* or *BTβ1*^A58T&R79E^ and examining the sensitivity of these strains to neonicotinoids using insecticide bioassays. Although homozygous strains not being able to be generated, the fly line expressing *BTβ1*^A58T&R79E^ exhibited significantly reduced sensitivity to all seven neonicotinoids tested compared to flies expressing wildtype *BTβ1*. Specifically, after 48 h feeding on insecticide concentrations of 75 mg/L and 150 mg/L, the mortality of the *BTβ1*^A58T&R79E^ expressing strain decreased 17.95% (p = 5.30 × 10^−3^) and 22.95% (p = 1.01 × 10^−4^) for imidacloprid compared to the *BTβ1* expressing strain (Fig 3B), for thiamethoxam the mortality decreased 33.84% (p = 1.20 × 10^−6^) and 30.66% (p = 1.01 × 10^−5^) (Fig 3C), for clothianidin 30.53% (p = 5.19 × 10^−5^) and 46.43% (p = 3.78 × 10^−5^) (Fig 3D), for acetamiprid 39.55% (p = 6.28 × 10^−6^) and 29.26% (p = 1.53 × 10^−4^) (Fig 3E), for dinotefuran 30.81% (p = 4.67 × 10^−5^) and 35.47% (p = 1.00 × 10^−4^) (Fig 3F), for nitenpyram 22.7% (p = 2.41 × 10^−4^) and 33.97% (p = 1.10 × 10^−4^) (Fig 3G). For thiacloprid, after feeding on concentrations of 200 and 400 mg/L the mortality of the *BTβ1*^A58T&R79E^ expressing strain decreased 29.07% (p = 3.33 × 10^−5^) and 33.04% (p = 4.21 × 10^−4^) at 48 h, compared to the *BTβ1* expressing strain (Fig 3H).

Previous study showed that changes in a nAChR subunit gene expression affect that of the other subunit gene expressions, resulting in changes of neonicotinoid sensitivity in *Drosophila* [34]. To confirm that whether the decreased sensitivities to neonicotinoids of transgenic flies in this study were caused by changes in expression levels of nicotinic subunit genes due to the introducing of these two mutations, relative expression levels of *Drosophila* nAChR subunit genes and *BTβ1* were compared between fly lines expressing *BTβ1* (WT) and *BTβ1*^A58T&R79E^ (MU). Results showed that no significant difference existing between the expression level of *Drosophila* nAChR genes and *BTβ1* in WT and MU fly lines (S4 Fig).

### Modelling of *BTβ1* suggests functional roles for R79E and A58T in neonicotinoid resistance

To examine the extent to which the two amino acids that are mutated in the R^#2^ strain are conserved across the diversity of insects, we screened the region encompassing these regions in the nAChR beta 1 subunit of more than 200 insect species. Without exception we found the A58 and R79 residues at these positions in all insects examined, revealing an exceptionally high level of conservation in insects (S5A Fig). In contrast the residues at these amino acid positions in mammals differed from insects, with uncharged residues, typically serine at position 58 and threonine at position 79, observed (S5B Fig).

**Fig 4:**
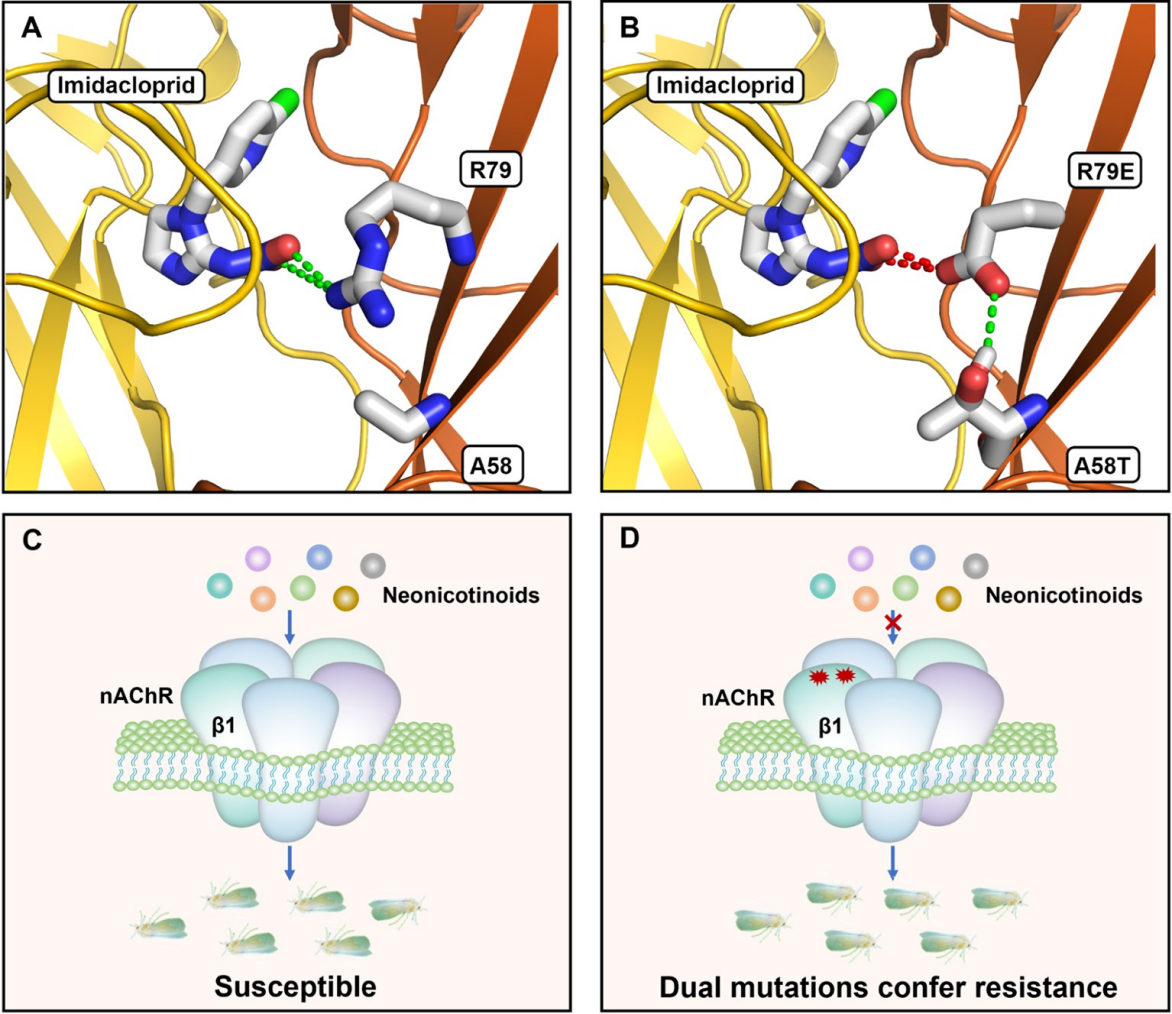
Homology models of wild-type and mutant *B*. *tabaci* nAChRs and a schematic of mutations in *BTβ1* conferring resistance to multiple neonicotinoids in *B*. *tabaci*. A docking prediction for imidacloprid is shown at the orthosteric site located between *α1* (yellow) and **(A)** *β1* (orange) or **(B)** *BTβ1*^A58T&R79E^ (orange) subunits. Green dashes show attractive electrostatic (A) or hydrogen bond (B) interactions and red dashes show a repulsive electrostatic interaction (B). **(C)** nAChRs containing wild-type *BTβ1* that allow the neonicotinoids-binding leads to the sensitivity of *B*. *tabaci*. **(D)** nAChRs containing *BTβ1*^A58T&R79E^ that electrostatically repulse neonicotinoids-binding leads to the resistance of *B*. *tabaci*.

A homology model of a *B*. *tabaci* α1β1 nAChR heteropentamer was generated to determine the relative positions of the A58 and R79 residues and to assess the effect their mutation may have on neonicotinoid binding. A58 and R79 were found to be located in close proximity on the β-subunit (Fig 4A), with 8 Å distance between their α-carbons. For docking studies at the orthosteric site, we used imidacloprid as an exemplar for neonicotinoids and allowed the side chain of R79 to flex during the docking run. A docking pose was generated that placed the positively-charged side chain of R79 in contact with the nitro tip of imidacloprid (Fig 4A). An attractive electrostatic interaction with R79 is predicted to result as each oxygen atom of the ligand’s nitro group carries a partial negative charge. In contrast, A58 was found to be too distant from the bound ligand to form a binding contact.

We introduced the A58T and R79E mutations into this nAChR model with docked imidacloprid. A conformation for the R79E side chain was chosen (Fig 4B) that closely matched the R79 rotamer of the wildtype nAChR (Fig 4A). Consequently, the negatively-charged carboxylate group of the R79E side chain is in contact with the imidacloprid nitro tip (Fig 4B) and, in this case, a repulsive electrostatic interaction is predicted to result. The A58T mutation introduces a larger side chain yet this also remains too distant from the binding site to form a ligand-binding contact. Instead, a rotamer was discovered for A58T that could allow the side-chain hydroxyl group to form a hydrogen bond with the R79E side chain (Fig 4B). The modelling indicates that, when engaged in a hydrogen bond with A58T, the side chain of R79E would be in a position to electrostatically repulse a neonicotinoid in the binding site. The reduced neonicotinoid sensitivity is the result of electrostatic repulsion by glutamate residue against the nitro group, and the threonine seemingly assists in such an interaction. Thus, we propose the A58T&R79E mutations in *BTβ1* confer resistance to neonicotinoids in *B*. *tabaci* by an electrostatic repulsion mechanism (Fig 4C and 4D).

## Discussion

Our data provide new insights into the nAChR gene family of whiteflies, the mutations in these genes that can lead to insecticide resistance, and the molecular determinants of neonicotinoid binding and selectivity.

We show that the nAChR gene family of *B*. *tabaci* encodes ten different subunits. When compared with the nAChR families of other insects [6–12], this number is the same as that of *D*. *melanogaster* (10 subunits) and *Anopheles gambiae* (10 subunits), but less than that of *Apis mellifera* (11 subunits), *Bombyx mori* (12 subunits), *Tribolium castaneum* (12 subunits), *Nasonia vitripennis* (16 subunits) and *Chilo suppressalis* (12 subunits). Importantly, the *B*. *tabaci* nAChR gene family comprises the core group of insect nAChRs [8], namely, *BTα1*–*4*, *BTα6–8* and *BTβ1*, which have orthologues in other insect species [6–12]. The *BTα5* gene is distinct from the *Diptera α5* subunit of *D*. *melanogaster* and *A*. *gambiae* [6,7], but is orthologous to the *α5* subunits of *A*. *mellifera*, *B*. *mori*, *T*. *castaneum*, *N*. *vitripennis* [8–12]. We also identified one divergent nAChR subunit gene in *B*. *tabaci*, *BTβ2*, that has no ortholog in other insect spieces [32]. The clustering of nAChR subunit genes in insect genomes may influence the co-expression and co-assembly of insect nAChR subunits [8,35]. As in *Drosophila*, *Dα1*, *Dα2* and *Dβ2* were clustering in genome [8], recent study has confirmed their co-expression in a *Drosophila* tissue [14], which further confirmed this assumption. In *B*. *tabaci*, only *BTα1* and *BTα2* were observed clustering in the *B*. *tabaci* genome, as seen for *A*. *mellifera* and *N*. *vitripenni* [8,11], suggesting they may be co-regulated and/or the subunits they encode co-assembled in certain nAChR subtypes.

Cloning of the ten *B*. *tabaci* nAChR subunit cDNAs identified alternative splice variants for both *BTα4* and *BTα6*. For *BTα4*, the alternative splicing occurred at exon 4, and provides alternate amino acid sequences in the B, E and Cys Loops. These are all functionally important regions, and thus the alternative splicing observed has the potential to alter characteristics of ligand-binding, receptor assembly and channel gating [36,37]. This alternative splicing is conserved in the insect nAChR families that have been annotated so far (S4 Table) [6–11], which further suggests an important conserved role in providing flexibility in optimizing the function of insect nAChRs for different conditions/cellular environments. In the case of *BTα6*, two alternative exons were identified for exon 8 that result in alternate sequences for the region between TM2 and TM3. The TM2-TM3 loop makes contact with the extracellular domain and the relative movement of these two regions enables coupling of ligand-binding to pore gating [37,38]. Sequence differences in the TM2-TM3 loop encoded by the alternative *BTα6* exons may therefore modify interactions with the extracellular domain and generate nAChRs with different gating kinetics. Similar splicing in exon 8 of *α6* has also been observed in other insects [6–9,11]. However, the exon 3 alternative splicing of *α6* was lost in *B*. *tabaci*, which have been observed in *D*. *melanogaster*, *A*. *gambiae*, *A*. *mellifera* and *T*. *castaneum* (S4 Table).

To date, RNA editing in insect nAChRs has been primarily detected in *D*. *melanogaster*, with *Dα5*, *Dα6*, *Dβ1* and *Dβ2* all found to be RNA edited [8]. In *B*. *tabaci*, only *BTα6* was found to have an RNA editing site, which occurs at an N-glycosylation site in loop E [8]. The presence of N-linked carbohydrate can affect cell trafficking and subunit assembly of nAChRs [39] and so RNA editing of the N-glycosylation site may be a pre-translational mechanism to control cell surface expression of this *BTα6* subunit. Interestingly, the same RNA editing site is also observed in *A*. *mellifera*, *N*. *vitripennis* and *T*. *castaneum* [8,9,11] suggestive of a conserved function in insects.

To date, neonicotinoid resistance in *B*. *tabaci* has been shown to primarily result from enhanced expression of metabolic enzymes such as the cytochrome P450 *CYP6CM1* [15,16,18,27,28]. However, in the current study we identified a *B*. *tabaci* MEAM1 strain that showed high resistance to neonicotinoids that was not impacted by inhibitors of the detoxification enzymes most commonly implicated in resistance. Through comparison of sequences of the nAChR subunit genes identified in this study from neonicotinoid resistant or susceptible *B*. *tabaci* strains, we identified two mutations, A58T and R79E, in *BTβ1* associated with resistance. We provide several lines of evidence that these mutations have a causal role in resistance. Firstly, neonicotinoid resistance was found to increase when a *B*. *tabaci* strain was made homozygous for the mutations, revealing a correlation between mutation frequency and level of resistance. Secondly, we demonstrated that while RNAi knockdown of wildtype *BTβ1* decreases the susceptibility of *B*. *tabaci* to neonicotinoids, consistent with our previous work [33], knockdown of *BTβ1*^A58T&R79E^ did not significantly decrease the sensitivity of the resistant strain to most neonicotinoid insecticides. This suggests that the characteristics of *BTβ1*^A58T&R79E^ and wildtype *BTβ1* differ in terms of their sensitivity to neonicotinoids. Finally transgenic *D*. *melanogaster* where the native nAChR *Dβ1* was replaced with *BTβ1*^A58T&R79E^ were significantly more resistant to neonicotinoids than flies where *Dβ1* were replaced with the wildtype *BTβ1* sequence, demonstrating that the mutations confer resistance *in vivo*. As extremely high resistance levels to several insecticides have been observed in homozygous mutant *B*. *tabaci* strain, the mortalities of transgenic fly expressing *BTβ1*^A58T&R79E^ only showed moderate decreases after feeding the neonicotinoids, compared to that of fly line expressing *BTβ1*. This can be caused by the heterozygous expression of *BTβ1*^A58T&R79E^ in *Drosophila* used for experiments, as the heterozygous R81T mutant *Drosophila* in previous study also exhibited relatively low resistance to neonicotinoids [13,40]. On the other hand, it has been recently found in *Drosophila* that the compensation of nAChR subunit genes exist, when expression level of one subunit gene was changed [34]. This significant finding proposed the assumption that whether the introducing of these two mutations can also cause this compensation and finally lead to the resistance phenotype of *Drosophila*. In fly lines we generated, we found no significant changes have been caused in relative expression level of other nAChR subunit genes by introducing these two mutations, which further indicated the resistance can be caused by the changes in pharmacological properties of *BTβ1*^A58T&R79E^, compared to that of *BTβ1* wild type.

To date, target-site mutations conferring neonicotinoid resistance have only been described in a handful of insect pests. These include the brown planthopper, *Nilaparvata lugens*, where imidacloprid resistance was associated with a mutation (Y151S) in two nAChR alpha subunits (*Nlα1* and *Nlα3*) [22]. Notably, neonicotinoid resistance in the aphids *Myzus persicae* and *Aphis gossypii* has been linked with the *β1* subunit R81T mutation, which is the equivalent loop D position as R79E identified in our study [23,24,41]. The causal role of the R81T mutation in neonicotinoid resistance has been previously demonstrated by both introducing this mutation into *D*. *melanogaster* using CRISPR:CAS genome editing and via heterologous expression of nAChRs with the mutation in *Xenopus laevis* oocytes [13,14,40]. The R81T mutation replaces a positively-charged side chain with an uncharged, polar side chain, resulting in a loss of interaction with the distinctive electronegative pharmacophore (nitro or cyano group) of neonicotinoid insecticides. The electrostatic nature of this interaction was first demonstrated electrophysiologically when the uncharged Q79 of chicken *α7* was mutated and the sensitivity of this homopentameric nAChR to imidacloprid significantly increased with Q79R but was virtually eliminated by the Q79E mutation [42]. The effect of these loop D mutations was minimal when tested with desnitro-imidacloprid, which identified the nitro group as the electrostatic contact point on imidacloprid. Thus, inhibition of insecticide binding due to electrostatic repulsion between the *B*. *tabaci β1* R79E side chain and neonicotinoid electronegative tip is the most likely molecular mechanism underlying the insecticide resistance that we observed with mutant *B*. *tabaci* flies.

The second mutation identified in our study, A58T, is located on loop G. A positively-charged residue occupying this position may contribute to neonicotinoid binding interactions [5,43,44]. However, in contrast to lysine or arginine, the threonine side chain of A58T is too short to directly contact a neonicotinoid at the orthosteric site. A hydrogen bond with R79E is possible and the role of A58T may therefore be to interact with and coordinate a conformation of the R79E side chain so that it is best positioned to inhibit neonicotinoid binding through electrostatic repulsion. An additional benefit of stabilizing this conformation of R79E is to inhibit the side chain from extending into the binding site, where it could engage in an electrostatic interaction with the positively-charged amine group of acetylcholine. In effect, A58T may act to sequester the R79E side chain to prevent it perturbing binding of the endogenous neurotransmitter. Taking into consideration that these two mutation sites distributed in different exons of *BTβ1*, they may not occur at the same period. And it can be supposed that the important role of interaction between 58T and 79E in physiological aspects could cause only the individuals carrying these dual mutations being selected in the field. Given the high frequency of the mutation in the mixed R^#1^ population without insecticide selection, it can also be indicated that there can be no significant fitness cost in this mutant strain. Further studies are required to investigate whether this mechanism explains the tight linkage between A58T and R79E and whether insects with the single R79E mutation exist or incur a fitness cost. Related to this, given the prevalence of P450-mediated neonicotinoid resistance in populations of *B*. *tabaci* worldwide [15,16,27–30], it will also be important to establish the resistance phenotype when R79E and A58T occur in a genetic background of enhanced P450 production. In particular, the practical impact of the two mechanisms in combination on the efficacy of foliar and systemic applications of neonicotinoids should be investigated.

In summary, we describe the nAChR family of *B*. *tabaci* and identify mutations in the *β1* subunit that confer neonicotinoid resistance. Our results are of applied importance for the control of a globally distributed, highly damaging crop pest and advance our understanding of neonicotinoid mode of action and selectivity.

## Materials and methods

### Insects and insecticides

The insect strains used in this study were *B*. *tabaci* MEAM1 strains, the S^#1^ strain was collected from Beijing, Haidian (39° 57’ N, 116° 17’ E) in 2000, the S^#2^ strain collected from Guangdong, Guangzhou (23° 09’ N, 113° 16’ E) in 2020 and the R^#1^ strain collected from Xinjiang, Urumqi (43° 82’ N, 87° 58’ E) in 2019. After collection all strains were maintained on cabbage in cages in a green house at 25°C without exposure to pesticides for more than 10 generations before used for experiment. The R^#2^ strain is homozygous fixed for the R79E and A58T mutations and was generated from R^#1^ as described below.

Imidacloprid (95%), clothianidin (98%), thiamethoxam (98%), dinotefuran (98%), acetamiprid (98%), thiacloprid (100 μg/mL in acetone) were supplied by Yuanye Bio-Tech (Shanghai, China); nitenpyram (95%) was supplied by Macklin Bio-Tech (Shanghai, China).

### Insecticide bioassays

Imidacloprid, thiamethoxam, clothianidin, acetamiprid were dissolved in acetone, and dinotefuran and nitenpyram were dissolved in water to a concentration of 10 g/L. Dissolved insecticides were diluted in insect diet (comprising 5% yeast extract and 30% sucrose, wt/vol dissolved in sterile water) [33]. A feeding device was used to expose *B*. *tabaci* to insecticides and was composed of a glass tube (length of 50 mm and diameter of 15 mm) and parafilm. One end of the tube was first covered with stretched parafilm, then 70 μL of insecticide containing diet was added to the surface of this parafilm. Another piece of stretched parafilm was then added onto the feeding liquid to form a feeding sachet. Approximately 25 individuals of *B*. *tabaci* (mixed sex) were then placed in this device, and the open end sealed with parafilm. A black plastic shell was used to cover the feeding device with only the feeding sachet side exposed to light to enhance feeding. After 48 h, individuals exhibiting no response to tapping were recorded as dead. LC_50_ (lethal concentration 50%) values were calculated using probit analysis in the POLO Plus 2.0 software (LeOra Software, USA).

Synergism assays were conducted using the same feeding sachet method as described above. The synergists TPP, PBO, DEM were mixed with insecticides at a concentration of 500 mg/L, 250 mg/L and 400 mg/L (the max concentration that will not lead to death of *B*. *tabaci* we tested). Two concentrations were conducted for each of the seven neonicotinoid insecticides, 250 and 125 mg/L for imidacloprid, 1500 and 600 mg/L for thiamethoxam, 900 and 200 mg/L for clothianidin, 700 and 175 mg/L for acetamiprid, 500 and 100 mg/L for dinotefuran, 675 and 270 mg/L for nitenpyram, 100 and 25 mg/L for thiacloprid. Number of deaths and total individuals were collected after feeding for 48h, and then used for mortality calculation. Three biological replicates were conducted for each experiment.

### RNA extraction and cDNA synthesis

Total RNA was extracted using TRIzol (Invitrogen) following the manufacturer’s protocol. Each sample comprised a pool of 50 adult *B*. *tabaci* (mixed sex). The concentration of each RNA sample was determined using a NanoDrop 2000c spectrophotometer (Thermo Fisher Scientific, Waltham, MA, USA). A HiScript 1st Strand cDNA Synthesis Kit (Vazyme, Nanjing, China) was used to synthesize cDNA from the extracted RNA. For cDNA used for gene cloning of *B*. *tabaci* nAChRs, the Oligo(dT) primer in this kit was used for reverse transcription. For cDNA used for qPCR detection, random hexamers provided in the kit were used.

### Identification of nAChR genes in the whitefly genome and phylogenetic analyses

Putative nAChR genes of *B*. *tabaci* were identified by screening the whitefly genome (http://www.whiteflygenomics.org/) [31] with the 11 nAChR cDNA sequences of *Apis mellifera* using the TBLASTN algorithm [8,45]. Primers to amplify the full-length of the 10 nAChR genes (*BTα1–8* and *BTβ1–2*) were then designed (S3 Table). The complete nucleotide sequence of these 10 *B*. *tabaci* nAChR genes were amplified using the 2×Phanta Max Master Mix (Vazyme, Nanjing, China), and then cloned using the pEASY-Blunt Cloning Kit (TransGen Biotech, Beijing, China). Positive clones were sent to Tsingke Bio-Tech (Beijing, China) for sequencing.

The cloned amino acid sequences of *B*. *tabaci* nAChRs, sequences of nAChR genes in other insects that have been annotated (*Drosophila melanogaster*, *Anopheles gambiae*, *Apis mellifera*, *Bombyx mori*, *Tribolium castaneum*, *Nasonia vitripennis*, *Chilo suppressalis*) [6–12] were aligned and used to create a phylogenetic tree using the neighbour-joining method with 1000 bootstrap replicates as implemented in MEGA 7.0.

### Construction of a *B*. *tabaci* strain homozygous for the R79E and A58T mutations

Primers were designed to amplify exon 2 and exon 3 regions of *BTβ1*, where the R79E and A58T mutations are located, using the *B*. *tabaci* genome as a reference (S3 Table). Single individuals of R^#1^ were transferred to individual 1.5 mL centrifuge tubes for PCR detection, using the MF848 M5 superluminal mix (Mei5 Biotech, Beijing, China) following the the manufacturer’s instructions. PCR products were sent to Tsingke Bio-Tech (Beijing, China) for sequencing. 60 individuals were screened for each *B*. *tabaci* strain. Heterozygous individuals in R^#1^ strain were found to be unreadable of their sequencing results, that can be caused by intron sequence differences between their mutant and wild type alleles (S3 Dataset).

To generate the *B*. *tabaci β1* homozygous mutant strain, 4^th^ instar nymphs of the R^#1^ strain were selected and transferred to individual 0.2 mL centrifuge tubes. Following emergence of adults, one female and one male adult were matched and transferred to a clean single leaf cotton plant placed in a small glass cage. 50 pairs were matched, and each cage was numbered. After one week, cages where *B*. *tabaci* parents survived were selected, and individuals transferred into clean 1.5 mL centrifuge tubes separately for mutation genotyping as described above. Cages in which the *B*. *tabaci* parents were found to carry the mutations in the homozygous form were reserved, and 20 individuals of their offspring were also genotyped. Finally, the remaining individuals from cages where all 20 offspring tested were found to be homozygous for the mutations were mixed together to generate the R^#2^ homozygous mutant strain.

### Quantitative real time PCR

For qPCR detection, primers were designed to generate products of 90–200 bp in size. Primers used for qPCR detection are listed in S3 Table. Reference genes were *RPL29* and *EF-1α* designed and used in a previous study [46]. QPCR reagent was supplied by Tiangen (Beijing, China), each reaction system comprised 2×SuperReal PreMix Plus (10 μL), Reference Dye (0.4 μL), primer (0.6 μL), cDNA (1 μL), and ddH_2_O (7.4 μL). The ABI Q3 real-time system (Thermo Fisher Scientific, USA) was used to perform qPCR reactions with the following temperature cycle: 95°C for 1 min, 40 cycles of 95°C for 5 s and 60°C for 15 s. Ct values of target genes were firstly normalized to that of reference gene to obtain the -ΔCt value in each sample. The -ΔCt value of treated group were normalized to that of control to obtain -ΔΔCt value, and then converted to fold data through 2^-ΔΔCt^ method, [47] two reference genes were used for analysis. Primers for qPCR detection of 13 genes that previously implicated in neonicotinoid resistance in whitefly [19] and *Drosophila* nAChR subunit genes [34] referred to primers used in the previous study. *Drosophila* reference genes were *β-actin* and *RPL32* [18]. Three individuals of fruit fly were used as a biological replicate, six biological replicates were conducted for each fly line.

### RNA interference

Double-stranded RNA of *BTβ1* and *EGFP* was prepared using the T7 RiboMAX Expression RNAi system (Promega, Madison, WI, USA) following the manufacturer’s instructions. Primers used for dsRNA preparation are listed in S3 Table. RNAi of *BTβ1* was performed using a dsRNA feeding concentration of 500 ng/μL for 48 h, according to the previously optimized protocol [33]. RNAi efficiency was assessed after feeding dsBTβ1 to the S^#1^ and R^#2^ strains using qPCR method compared to the control group fed dsEGFP.

After feeding on dsRNA whiteflies were transferred to feeding devices for determination of changes in susceptibilities to the seven neonicotinoid insecticides. The feeding concentration selected corresponded to the LC_50_ values of each insecticide. For the S^#1^ strain, the feeding concentration for imidacloprid was 5 mg/L; for thiamethoxam, clothianidin, dinotefuran, nitenpyram, acetamiprid 2.5 mg/L; for thiacloprid 1 mg/L. For the R^#2^ strain, the feeding concentration used for imidacloprid was 50 mg/L; for thiamethoxam, clothianidin, dinotefuran, acetamiprid 200 mg/L was used; for thiacloprid and nitenpyram 20 mg/L and 100 mg/L were used respectively. Whitefly mortality was recorded, and data was compared between the dsBTβ1 and dsEGFP group in the S^#1^ and R^#2^ strains. Three biological replicates were conducted for each condition.

### Creation of transgenic *Drosophila melanogaster*

Wild-type *BTβ1* and *BTβ1*^A58T&R79E^ were amplified and cloned into the pJFRC28-10XUAS-IVS-GFP-p10 plasmid (Addgene #36431). Using the PhiC31 system, clones were individually transformed into the germ line of a *D*. *melanogaster* nAChR*β1*-attP strain (heterozygous *Dβ1* knock-out, a gift from Dr. Yi Rao [13], Peking University). The transgenic lines obtained were balanced, and the integration of *BTβ1* was confirmed by PCR and sequencing using the Phanta Max Super-Fidelity DNA Polymerase (Vazyme) with the primers detailed in S3 Table. Virgin females of the Tub-GAL4[w1118; P{w(+mC) = tubP-GAL4}2/CyO] strain were crossed with males of the UAS-*BTβ1* strain (FunGene). Homozygous *Dβ1* knock-out individuals were selected through their offspring by individuals with a small haltere, however, it was not possible to construct a homozygous knock out strain despite repeated attempts. The individuals used for insecticide bioassays were a mix of heterozygous and homozygous flies. Several concentrations of each insecticide were overlaid onto 2% agar containing 1% sucrose in standard *Drosophila* vials and allowed to dry overnight at room temperature. Ten to 20 adult flies (2 to 5 days after eclosion) were then added to each vial, and mortality was assessed after 48 hours. Four replicates were carried out for each concentration. Control mortality was assessed using vials containing agar/sucrose minus insecticide.

### Homology modelling and ligand docking

The cryo-electron microscopy structure of the human α3β4 nAChR in complex with nicotine [48] (PDB entry 6PV7) provided the template for homology modelling of the *B*.*tabaci* α1β1 heteropentamer. Sequences of α-type subunit gene (human α3 and *B. tabaci* α1) and non-α subunit gene (human β4 and *B. tabaci* β1) were aligned respectively using Clustal Omega, and 50 homology models were generated using MODLLER v10.4 [49]. Generated models were ranked according to the internal scoring function of MODELLER, and the top-scoring model was assessed in Swiss-PdbViewer [50] to confirm the absence of stereochemical problems. Swiss-PdbViewer was also used to introduce the R79E and A58T mutations and to subsequently apply 30 steps of steepest descent followed by 50 steps of conjugate gradient energy minimization to the mutant model.

A structure file for imidacloprid was generated *ab initio* using MarvinSketch v19.22 of the ChemAxon suite (https://chemaxon.com/). AutoDockTools v1.5.7 [51] was used to define rotatable bonds and merge nonpolar hydrogens and Autodock Vina v1.2.3 [52] was used to generate docking predictions for imidacloprid in a grid of 20 X 20 X 20 points with 1 Å spacing positioned on the orthosteric site of the wild-type nAChR model. The side chains of loop D R79 and loop E L139 were allowed to flex during the docking to allow interaction between R79 and the neonicotinoid ligand. Figures were produced using PyMOL (https://pymol.org).

### Data analysis

The statistical significance of differences between samples was analyzed by Student’s t test and analysis of variance (ANOVA) with Tukey’s post hoc test (GraphPad 7.0). All quantitative data are reported as means ± SEM from at least three independent experiments.

## Supporting information

S1 Fig Amino acid sequences of nAChR subunits in *B*. *tabaci*. Adjacent cysteine residues in Loop-C that are characteristic of *α* or non-*α* subunits are boxed.
(DOCX)

S2 Fig Amino acid sequence identity between nAChR subunits of *Apis melifera* and *Bemisia tabaci*. Sequences were aligned and the degree of sequence similarity in percentage were generated by DNAMAN 8.
(DOCX)

S3 Fig Comparison of relative expression level of genes that previously implicated in neonicotinoid resistance in whitefly between S^#2^ and R^#2^ strain. Values are means of six biological replicates (*n* = 6). Each bar represents the mean ± standard deviation.
(DOCX)

S4 Fig Comparison of relative expression level of nAChR subunit genes between *Drosophila* strains expressing *BTβ1* (WT) and *BTβ1*^A58T&R79E^ (MU). Values are means of six biological replicates (*n* = 6), each bar represents the mean ± standard deviation.
(DOCX)

S5 Fig **(A)** Conservation of the 58A and 79R site in 200 insect spieces. **(B)** Conservation of the 58S and 79T in vertebrate nAChR. These sites are boxed and labled with asterisks above the alignment.
(DOCX)

S1 Table Chromosal location of nAChR subunit genes in *B*. *tabaci*. (DOCX)

S2 Table Log-dose probit-mortality data for *B*. *tabaci* strains in response to different neonicotinoid insecticides. (DOCX)

S3 Table Oligonucleotide primers used in this study. (DOCX)

S4 Table Comparison of the nAChR gene family in select insects. (DOCX)

S1 Dataset Analysis of the sequences of eight nAChR *α* subunits and two *β* subunits in the S^#1^, S^#2^ and R^#1^ *B*. *tabaci* strains. (DOCX)

S2 Dataset Detection of the A to I editing site in *BTα6* in the S^#2^ and R^#2^ *B*. *tabaci* strains. (DOCX)

S3 Dataset Detection of the A58T mutation in exon 2 and the R79E mutation in exon 3 of *BTβ1* in the S^#1^, S^#2^, R^#1^ and R^#2^ *B*. *tabaci* strains. (DOCX)
