# Supplementary material for: Dual mutations in the whitefly nicotinic acetylcholine receptor β1 subunit confer target-site resistance to multiple neonicotinoid insecticides
Source: PLoS Genet. 2024 Feb 20;20(2):e1011163. doi: 10.1371/journal.pgen.1011163 (PMC10906874; doi:10.1371/journal.pgen.1011163)
Supplement: S1 Fig — Adjacent cysteine residues in Loop-C that are characteristic of α or non-α subunits are boxed. (DOCX) [file pgen.1011163.s001.docx]

**S1 Fig.** Amino acid sequences of nAChR subunits in *B. tabaci*. Adjacent cysteine residues in Loop-C that are characteristic of *α* or non-*α* subunits are boxed.
