# Supplementary material for: Dual mutations in the whitefly nicotinic acetylcholine receptor β1 subunit confer target-site resistance to multiple neonicotinoid insecticides
Source: PLoS Genet. 2024 Feb 20;20(2):e1011163. doi: 10.1371/journal.pgen.1011163 (PMC10906874; doi:10.1371/journal.pgen.1011163)
Supplement: S2 Fig — Sequences were aligned and the degree of sequence similarity in percentage were generated by DNAMAN 8. (DOCX) [file pgen.1011163.s002.docx]

**S2 Fig.** Amino acid sequence identity between nAChR subunits of *Apis melifera* and *Bemisia tabaci*. Sequences were aligned and the degree of sequence similarity in percentage were generated by DNAMAN 8.
