# Supplementary material for: Dual mutations in the whitefly nicotinic acetylcholine receptor β1 subunit confer target-site resistance to multiple neonicotinoid insecticides
Source: PLoS Genet. 2024 Feb 20;20(2):e1011163. doi: 10.1371/journal.pgen.1011163 (PMC10906874; doi:10.1371/journal.pgen.1011163)
Supplement: S3 Fig — Values are means of six biological replicates (n = 6). Each bar represents the mean ± standard deviation. (DOCX) [file pgen.1011163.s003.docx]

**S3 Fig.** Comparison of relative expression level of genes that previously implicated in neonicotinoid resistance in whitefly between S^#2^ and R^#2^ strain. Values are means of six biological replicates (*n* = 6). Each bar represents the mean ± standard deviation.
