# Supplementary material for: Dual mutations in the whitefly nicotinic acetylcholine receptor β1 subunit confer target-site resistance to multiple neonicotinoid insecticides
Source: PLoS Genet. 2024 Feb 20;20(2):e1011163. doi: 10.1371/journal.pgen.1011163 (PMC10906874; doi:10.1371/journal.pgen.1011163)
Supplement: S4 Fig — Values are means of six biological replicates (n = 6), each bar represents the mean ± standard deviation. (DOCX) [file pgen.1011163.s004.docx]

**S4 Fig.** Comparison of relative expression level of nAChR subunit genes between *Drosophila* strains expressing *BTβ1* (WT) and *BTβ1^A58T&R79E^* (MU). Values are means of six biological replicates (*n* = 6), each bar represents the mean ± standard deviation.
