# Supplementary material for: Dual mutations in the whitefly nicotinic acetylcholine receptor β1 subunit confer target-site resistance to multiple neonicotinoid insecticides
Source: PLoS Genet. 2024 Feb 20;20(2):e1011163. doi: 10.1371/journal.pgen.1011163 (PMC10906874; doi:10.1371/journal.pgen.1011163)
Supplement: S5 Fig — (A) Conservation of the 58A and 79R site in 200 insect spieces. (B) Conservation of the 58S and 79T in vertebrate nAChR. These sites are boxed and labled with asterisks above the alignment. (DOCX) [file pgen.1011163.s005.docx]

**S5 Fig. (A)** Conservation of the 58A and 79R site in 200 insect spieces. **(B)** Conservation of the 58S and 79T in vertebrate nAChR. These sites are boxed and labled with asterisks above the alignment.
