# Supplementary material for: Dual mutations in the whitefly nicotinic acetylcholine receptor β1 subunit confer target-site resistance to multiple neonicotinoid insecticides
Source: PLoS Genet. 2024 Feb 20;20(2):e1011163. doi: 10.1371/journal.pgen.1011163 (PMC10906874; doi:10.1371/journal.pgen.1011163)
Supplement: S1 Table — (DOCX) [file pgen.1011163.s006.docx]

**S1 Table:** Chromosal location of nAChR subunit genes in *B. tabaci*.

| **Chromosomal number** | **Gene**  **name** | **Number of exons** | **Chromosomal location** | **Length** |
| --- | --- | --- | --- | --- |
| 05 | *BTα1* | 8 | 36,147,312-36,255,681 | 108,370 |
|  | *BTα2* | 8 | 36,502,374-36,587,680 | 85,307 |
| 04 | *BTα3* | 12 | 56,986,476-57,067,660 | 81,185 |
| 09 | *BTα4* | 11 | 28,789,361-29,078,859 | 289,499 |
| 01 | *BTα5* | 9 | 41,964,183-42,201,163 | 236,981 |
| 06 | *BTα6* | 11 | 3,070,506-3,588,958 | 518,453 |
|  | *BTβ1* | 7 | 19,070,280-19,089,726 | 19,447 |
|  | *BTα7* | 10 | 37,178,763-37,462,108 | 283,346 |
| 02 | *BTβ2* | 9 | 24,328,187-24,343,553 | 15,367 |
|  | *BTα8* | 12 | 47,206,584-47,219,036 | 12,453 |
