# Supplementary material for: Dual mutations in the whitefly nicotinic acetylcholine receptor β1 subunit confer target-site resistance to multiple neonicotinoid insecticides
Source: PLoS Genet. 2024 Feb 20;20(2):e1011163. doi: 10.1371/journal.pgen.1011163 (PMC10906874; doi:10.1371/journal.pgen.1011163)
Supplement: S2 Table — (DOCX) [file pgen.1011163.s007.docx]

**S2 Table:** Log-dose probit-mortality data for B. tabaci strains in response to different neonicotinoid insecticides.

| **Population** | **Insecticide** | **^1^N** | **Slope (± SE)** | **LC_50_ (mg L^-1^)** | **(95% ^2^FL)** | **^3^RR** |
| --- | --- | --- | --- | --- | --- | --- |
| S^#1^ | imidacloprid | 351 | 1.54( ± 0.15) | 5.40^a^ | 4.78-6.13 | - |
|  | clothianidin | 354 | 3.84( ± 0.39) | 2.65^a^ | 2.48-2.82 | - |
|  | thiacloprid | 654 | 1.16( ± 0.09) | 0.55^a^ | 0.48-0.63 | - |
|  | nitenpyram | 321 | 1.12( ± 0.04) | 0.54^a^ | 0.50-0.58 | - |
|  | dinotefuran | 457 | 1.70( ± 0.21) | 2.02^a^ | 1.75-2.3 | - |
|  | thiamethoxam | 514 | 1.37( ± 0.08) | 2.02^a^ | 1.84-2.2 | - |
|  | acetamiprid | 632 | 1.32( ± 0.11) | 1.44^a^ | 1.25-1.64 | - |
| S^#2^ | imidacloprid | 468 | 1.61( ± 0.09) | 4.93^a^ | 4.57-5.32 | 0.91 |
|  | clothianidin | 643 | 3.79( ± 0.29) | 3.08^b^ | 2.95-3.21 | 1.16 |
|  | thiacloprid | 267 | 1.45( ± 0.08) | 0.60^a^ | 0.55-0.65 | 1.09 |
|  | nitenpyram | 293 | 1.06( ± 0.06) | 0.44^a^ | 0.4-0.5 | 0.81 |
|  | dinotefuran | 712 | 1.98( ± 0.17) | 2.50^a^ | 2.29-2.73 | 1.24 |
|  | thiamethoxam | 532 | 1.76( ± 0.12) | 4.48^b^ | 4.09-4.89 | 2.22 |
|  | acetamiprid | 543 | 1.47( ± 0.11) | 1.96^b^ | 1.75-2.2 | 1.36 |
| R^#1^ | imidacloprid | 644 | 1.37( ± 0.14) | 47.96^b^ | 40.95-55.84 | 8.88 |
|  | clothianidin | 346 | 1.77( ± 0.16) | 395.9^c^ | 362.4-438 | 149.4 |
|  | thiacloprid | 642 | 1.34( ± 0.16) | 40.15^b^ | 33.41-47.96 | 72.46 |
|  | nitenpyram | 590 | 1.12( ± 0.06) | 49.16^b^ | 44.08-54.75 | 91.72 |
|  | dinotefuran | 307 | 0.99( ± 0.11) | 201^b^ | 168.3-249.1 | 99.41 |
|  | thiamethoxam | 471 | 2.21( ± 0.22) | 210.9^c^ | 196-226.2 | 104.51 |
|  | acetamiprid | 298 | 2.01( ± 0.26) | 274.1^c^ | 234.4-317.5 | 191.01 |
| R^#2^ | imidacloprid | 408 | 1.75( ± 0.10) | 88.76^c^ | 82.93-95.04 | 16.44 |
|  | clothianidin | 764 | 2.00( ± 0.13) | 472.1^d^ | 443.5-502.1 | 178.15 |
|  | thiacloprid | 279 | 1.32( ± 0.09) | 57.02^c^ | 51.17-63.55 | 102.91 |
|  | nitenpyram | 468 | 1.71( ± 0.09) | 78.27^c^ | 72.29-84.76 | 146.02 |
|  | dinotefuran | 579 | 1.12( ± 0.10) | 266^b^ | 228.1-314.9 | 131.55 |
|  | thiamethoxam | 469 | 1.90( ± 0.08) | 276.4^d^ | 265-288.5 | 136.97 |
|  | acetamiprid | 530 | 2.18( ± 0.09) | 324.9^c^ | 310.4-340.1 | 226.41 |

^1^N = Number of *B.tabaci* used in each bioassay.

^2^FL = Fiducial limit.

^3^RR (Resistance Ratio) = LC_50_ of the sample strains/LC_50_ of strain S^#1^.

Different letters on LC_50_ values represent significant difference between populations for each insecticide (*P* < 0.05).
