# Supplementary material for: Dual mutations in the whitefly nicotinic acetylcholine receptor β1 subunit confer target-site resistance to multiple neonicotinoid insecticides
Source: PLoS Genet. 2024 Feb 20;20(2):e1011163. doi: 10.1371/journal.pgen.1011163 (PMC10906874; doi:10.1371/journal.pgen.1011163)
Supplement: S3 Table — (DOCX) [file pgen.1011163.s008.docx]

**S3 Table.** Oligonucleotide primers used in this study.

| **Primer name** | **Usage** | **Primer squence 5′-3′^a^** | **Annealing temperature (°C)** | **Product (bp)** | **Amplification efficiencies (%)** |
| --- | --- | --- | --- | --- | --- |
| BTα1 | Full-Length | F-ATGCTGGGCGTGGTTAGAATG | 60 | 1686 | - |
|  |  | R-ATCCTCCTCGGGGCCCAT |  |  |  |
| BTα2 | Full-Length | F-ATGTGCTTCCGGGAGTGGC | 60 | 1668 | - |
|  |  | R-AAAATCGGAAGTGACGTCGGG |  |  |  |
| BTα3 | Full-Length | F-ATGAGGATAATTTACTGGATAATCGTTGT | 60 | 1707 | - |
|  |  | R-GAGTCGTGGTAGTAACTTGCGGC |  |  |  |
| BTα4 | Full-Length | F-ATGTGTGTGGTTGAGACGGTGC | 60 | 1692 | - |
|  |  | R-CTATTTCGGTGGCGGGCA |  |  |  |
| BTα5 | Full-Length | F-ATGCGCCTGTTGAGTGACAATC | 60 | 1452 | - |
|  |  | R-CGGGCCGTATGGGGATC |  |  |  |
| BTα6 | Full-Length | F-ATGATTCGGGCCGACTCCTAT | 60 | 1479 | - |
|  |  | R-CTGGACGATGATGTGCGGG |  |  |  |
| BTα7 | Full-Length | F-ATGGTGCGGATCGGTGTTG | 60 | 1515 | - |
|  |  | R-GGATGTTGACGATAATATGCGGG |  |  |  |
| BTα8 | Full-Length | F-ATGCTCTTCGTTTTTGCTTTAATCC | 60 | 1602 | - |
|  |  | R-AAGTGCATCTAAAATTTTGTCTCGGT |  |  |  |
| BTβ1 | Full-Length | F-ATGAAAACGTCGCTAGTGGCTACT | 60 | 1524 | - |
|  |  | R-CTTTCCACGGTAGATCTCAATTATTCTAT |  |  |  |
| BTβ2 | Full-Length | F-ATGATTGCTAAACGTTTTTTGGATCT  R-TTAGTAAAAACACTTTATCAAAATGATGAGGT | 60 | 1401 | - |
| BTα6-ex5 | A to I site detect | F-ATTCGACGGCACGTTTCACA | 60 | 173 | - |
|  |  | R-TTCATATCGCACTTCTGGTCGTC |  |  |  |
| BTβ1-ex2 | A58T detect | F-TTTCAGGCCTGTGTTCAGAGGA | 60 | 238 | - |
|  |  | R-TTTGTAAGACTCCGTTTTTATTTCGA |  |  |  |
| BTβ1-ex3 | R79E detect | F-ATTCTATCCTGGCGTAACTCGC | 60 | 386 | - |
|  |  | R-TGATGTACTTATGGCTGCTGCTACTA |  |  |  |
| BTβ1-ds | dsRNA | F-taatacgactcactataggTTCCTCCTGCCATTTACCAG  R-taatacgactcactataggTCGGCAGGATTAAATTGACC | 57 | 327 | - |
| EGFP | dsRNA | F-taatacgactcactatagggGAGACAGTGCTTCAGCCGCTAC  R-taatacgactcactatagggGAGAGTTCACCTTGATGCCGTTC | 57 | - | - |
| BTβ1-q | qPCR | F-CGTCTGGTTGCGACTGGTATG  R-TTCGTAGTTACCATCGGCATTGT | 60 | 145 | 99 |
| EF1α | qPCR | F-TAGCCTTGTGCCAATTTCCG  R-CCTTCAGCATTACCGTCC | 60 | 110 | 103 |
| RPL29 | qPCR | F-TCGGAAAATTACCGTGAG  R-GAACTTGTGATCTACTCCTCTCGTG | 60 | 144 | 101 |
| HSP | Transgenic fly detect | F-AAGTAACCAGCAACCAAGTA | 60 | 1962 | - |
|  |  | R-GCCACTAGCTCGCTATACACT |  |  |  |

^a^F, forward primer; R, reverse primer
