# Supplementary material for: Dual mutations in the whitefly nicotinic acetylcholine receptor β1 subunit confer target-site resistance to multiple neonicotinoid insecticides
Source: PLoS Genet. 2024 Feb 20;20(2):e1011163. doi: 10.1371/journal.pgen.1011163 (PMC10906874; doi:10.1371/journal.pgen.1011163)
Supplement: S4 Table — (DOCX) [file pgen.1011163.s009.docx]

**S4 Table:** Comparison of the nAChR gene family in select insects.

| **Insect** | **Number of subunits** | **Number of Alternative exons** | | | | | **A to I editing** | **Subunit clustering in Genome** |
| --- | --- | --- | --- | --- | --- | --- | --- | --- |
|  |  | ***α4*** | ***α4*** | ***α6*** | ***α6*** | ***α8*** |  |  |
|  |  | **exon 3** | **exon 4** | **exon 3** | **exon 8** | **exon 7** |  |  |
| *D. melanogaster* | 10 | 1 | 2 | 2 | 3 | 1 | *α5, α6, β1, β2* | *α1, α2, β2* |
| *A.*  *gambiae* | 10 | 1 | 2 | 2 | 3 | 1 | None | *α1, α2, α8* |
|  |  |  |  |  |  |  |  | *α7, β1* |
| *A.*  *mellifera* | 11 | 1 | 2 | 2 | 2 | 1 | *α6* | *α1, α2* |
|  |  |  |  |  |  |  |  | *α7, β1* |
|  |  |  |  |  |  |  |  | *α9, β2* |
| *B.*  *mori* | 12 | 2 | 2 | 1 | 1 | 2 | None | *-* |
| *T.*  *castaneum* | 12 | 1 | 2 | 2 | 3 | 1 | *α6* | *α7, β1* |
|  |  |  |  |  |  |  |  | *α8, α11* |
| *N.*  *vitripennis* | 16 | 1 | 2 | 1 | 3 | 1 | *α6* | *α1, α2, α5* |
|  |  |  |  |  |  |  |  | *β2, β3* |
|  |  |  |  |  |  |  |  | *α11, α12* |
| *B.*  *tabaci* | 10 | 1 | 2 | 1 | 2 | 1 | *α6* | *α1, α2* |
