# Supplementary material for: Dual mutations in the whitefly nicotinic acetylcholine receptor β1 subunit confer target-site resistance to multiple neonicotinoid insecticides
Source: PLoS Genet. 2024 Feb 20;20(2):e1011163. doi: 10.1371/journal.pgen.1011163 (PMC10906874; doi:10.1371/journal.pgen.1011163)
Supplement: S1 Dataset — (DOCX) [file pgen.1011163.s010.docx]

**Analysis of the *BTα1* sequences in the S^#1^, S^#2^ and R^#1^ *B. tabaci*** **strains**

**Analysis of the *BTα2* sequences in the S^#1^, S^#2^ and R^#1^ *B. tabaci*** **strains**

**Analysis of the *BTα3* sequences in the S^#1^, S^#2^ and R^#1^ *B. tabaci*** **strains**

**Analysis of the *BTα4* sequences in the S^#1^, S^#2^ and R^#1^ *B. tabaci*** **strains**

**Analysis of the *BTα5* sequences in the S^#1^, S^#2^ and R^#1^ *B. tabaci*** **strains**

**Analysis of the *BTα6* sequences in the S^#1^, S^#2^ and R^#1^ *B. tabaci*** **strains**

**Analysis of the *BTα7* sequences in the S^#1^, S^#2^ and R^#1^ *B. tabaci*** **strains**

**Analysis of the *BTα8* sequences in the S^#1^, S^#2^ and R^#1^ *B. tabaci*** **strains**

**Analysis of the *BTβ1* sequences in the S^#1^, S^#2^ and R^#1^ *B. tabaci*** **strains**

**Analysis of the *BTβ2* sequences in the S^#1^, S^#2^ and R^#1^ *B. tabaci*** **strains**
