# Supplementary material for: Dual mutations in the whitefly nicotinic acetylcholine receptor β1 subunit confer target-site resistance to multiple neonicotinoid insecticides
Source: PLoS Genet. 2024 Feb 20;20(2):e1011163. doi: 10.1371/journal.pgen.1011163 (PMC10906874; doi:10.1371/journal.pgen.1011163)
Supplement: S2 Dataset — (DOCX) [file pgen.1011163.s011.docx]

**Dataset S2.** Detection of the A to I editing site of *BTα6* in the S^#2^ and R^#2^ *B. tabaci* strains.
