# Supplementary material for: Dual mutations in the whitefly nicotinic acetylcholine receptor β1 subunit confer target-site resistance to multiple neonicotinoid insecticides
Source: PLoS Genet. 2024 Feb 20;20(2):e1011163. doi: 10.1371/journal.pgen.1011163 (PMC10906874; doi:10.1371/journal.pgen.1011163)
Supplement: S3 Dataset — (DOCX) [file pgen.1011163.s012.docx]

**Dataset S3.** Detection of the A58T mutation in exon 2 and the R79E mutation in exon 3 of *BTβ1* in the S^#1^, S^#2^, R^#1^ and R^#2^ *B. tabaci* strains.

**Table. Frequency of 58T&79E present in *B. tabaci* S^#1^, S^#2^, R^#1^ and R^#2^ strains.**

| **Strain** | **N^a^** | **Number of Sequencing Results** | | | | | **Number of Alleles** | | |
| --- | --- | --- | --- | --- | --- | --- | --- | --- | --- |
|  |  | **Homozygous** | | **Heterozygous** | | |  |  |  |
|  |  | **58A&79R** | **58T&79E** | | **-** | **58T&79E** | | **total** | **Frequency** |
| S^#1^ | 60 | 60 | 0 | | 0 | 0 | | 120 | 0 |
| S^#2^ | 60 | 60 | 0 | | 0 | 0 | | 120 | 0 |
| R^#1^ | 68 | 7 | 53 | | 8 | 114 | | 136 | 83.82 |
| R^#2^ | 60 | 0 | 60 | | 0 | 120 | | 120 | 100 |

^a^N = Number of *B.tabaci* used for detection.
